# Impact of Premature Senescence on Radiosensitivity Measured by High Throughput Cell-Based Assays

**DOI:** 10.3390/ijms18071460

**Published:** 2017-07-06

**Authors:** Razmik Mirzayans, Bonnie Andrais, David Murray

**Affiliations:** Department of Oncology, University of Alberta, Cross Cancer Institute, Edmonton, AB T6G 1Z2, Canada; bonnie.andrais@ahs.ca (B.A.); david.murray5@ahs.ca (D.M.)

**Keywords:** ionizing radiation, p53 signaling, premature senescence, viability, proliferation, apoptosis, colony forming ability, MTT, XTT, CellTiter-Blue

## Abstract

In most p53 wild-type human cell types, radiosensitivity evaluated by the colony formation assay predominantly reflects stress-induced premature senescence (SIPS) and not cell death (*Int. J. Mol. Sci.* 2017, 18, 928). SIPS is a growth-arrested state in which the cells acquire flattened and enlarged morphology, remain viable, secrete growth-promoting factors, and can give rise to tumor-repopulating progeny. The impact of SIPS on radiosensitivity measured by short-term assays remains largely unknown. We report that in four p53 wild-type human solid tumor-derived cell lines (HCT116, SKNSH, MCF7 and A172): (i) the conventional short-term growth inhibition assay (3 days post-irradiation) generates radiosensitivity data comparable to that measured by the laborious and time-consuming colony formation assay; (ii) radiation dose-response curves obtained by multiwell plate colorimetric/fluorimetric assays are markedly skewed towards radioresistance, presumably reflecting the emergence of highly enlarged, growth-arrested and viable cells; and (iii) radiation exposure (e.g., 8 Gy) does not trigger apoptosis or loss of viability over a period of 3 days post-irradiation. Irrespective of the cell-based assay employed, caution should be exercised to avoid misinterpreting radiosensitivity data in terms of loss of viability and, hence, cell death.

## 1. Introduction

Activation of the p53 signaling pathway by ionizing radiation was originally proposed to result either in cell cycle checkpoint activation to facilitate DNA repair and promote survival, or in apoptotic cell death (e.g., [1]). Based on this two-armed model, radiosensitivity assessed by multiwell plate colorimetric/fluorimetric assays (e.g., MTT, XTT, CellTiter-Blue) would be expected to reflect cell death. Accordingly, the outcomes of such radiosensitivity (and indeed chemosensitivity) assays are often interpreted to reflect loss of viability and hence cell death. However, such an assumption may be misleading. As extensively reviewed recently [2,3,4], a large body of evidence has established that the primary response triggered by moderate doses of ionizing radiation (between 2 and 8 Gy) in solid tumor-derived cell lines is in fact a sustained proliferation block, and not apoptosis. The proliferation block predominantly reflects stress-induced premature senescence (SIPS) in cells that express wild-type p53 [5,6,7,8,9].

SIPS is a sustained growth arrested state characterized by the acquisition of flattened and enlarged cell morphology in cells that retain viability for a long time (months) post-treatment and acquire the ability to secrete factors that can promote proliferation and invasiveness in cell culture models and tumor development in vivo [10,11]. In addition, under some conditions, cancer cells undergoing SIPS can give rise to stem cell-like progeny, thereby contributing to cancer relapse following therapy [3,12,13]. Thus, although cancer cells undergoing SIPS might be scored as “dead” in the short-term (colorimetric) and long-term (colony formation) assays in vitro, and can potentially contribute to tumor-growth delay in animal model studies, the senescence-like growth-arrested response (tumor dormancy) may in fact represent an important cell-survival mechanism, ultimately resulting in tumor regrowth and disease recurrence [2,3,14,15].

The p21^WAF1^ (p21; also called CDKN1A) protein is the founding member of the CIP/KIP family of cyclin-dependent kinase (CDK) inhibitors [16,17]. It is a p53 transcriptional target that plays a pivotal role in the DNA damage surveillance network, not only by activating early cell cycle checkpoints, but also through suppressing apoptosis (e.g., by inhibiting caspases), switching on the SIPS program, and contributing to the maintenance of the sustained growth arrested response, a hallmark of SIPS [18,19]. Consistent with these multifunctional properties of p21, radiosensitivity as measured by the colony formation assay in most cell types that express wild-type p53 (e.g., skin fibroblast strains; solid tumor-derived cell lines) is shown to be largely, if not totally, associated with growth arrest through p21-mediated SIPS [2,3,6,7,20].

The purpose of the present study was to determine the impact of SIPS on radiosensitivity when evaluated by short-term tests in the commonly-used p53 wild-type cancer cell lines HCT116 (colon carcinoma), MCF7 (breast carcinoma), SKNSH (neuroblastoma), and A172 (malignant glioma). These cell lines are known to respond to moderate doses of ionizing radiation (e.g., 8 Gy) by exhibiting sustained nuclear accumulation of p21, coupled with growth arrest through SIPS. We demonstrate that radiosensitivity as measured by multiwell plate colorimetric/fluorimetric assays is markedly skewed towards radioresistance when compared to that measured by growth inhibition (3 days post-irradiation) and colony formation (10 days post-irradiation) assays, and that the response measured by these cell-based assays reflects growth inhibition (SIPS) and not apoptosis or loss of viability.

## 2. Results and Discussion

### 2.1. Colony Formation and Growth Inhibition Assays Indicate a Comparable Degree of Radiosensitivity in p53 Wild-Type Cancer Cells

The colony formation assay (also called clonogenic survival) for assessment of radiosensitivity in cultured human cells was developed by Puck and Marcus over half a century ago [21]. It has since been used for studying the effectiveness of different agents (radiation, chemicals, or a combination of the two) on the survival and proliferation of various mammalian cell types. The typical assay for evaluating radiosensitivity involves preparing a single-cell suspension from a monolayer culture, plating out cells at very low densities (e.g., 300 cells per 60-mm tissue culture dish), exposing them to graded doses of ionizing radiation, and incubating them for 10–14 days to allow the formation of macroscopic colonies, each comprised of aggregates of at least 50 cells. The reduction in macroscopic colony number in irradiated compared to sham-treated samples reflects the degree of radiosensitivity, i.e., loss of reproductive potential. We have performed pilot experiments with skin fibroblast strains and cancer cell lines and found that irradiating the cells either prior to seeding them at cloning densities or shortly afterwards (within ~4 h post-plating for cancer cell lines [22]) does not impact on the outcome of the assay (unpublished observations).

**Figure 1:**
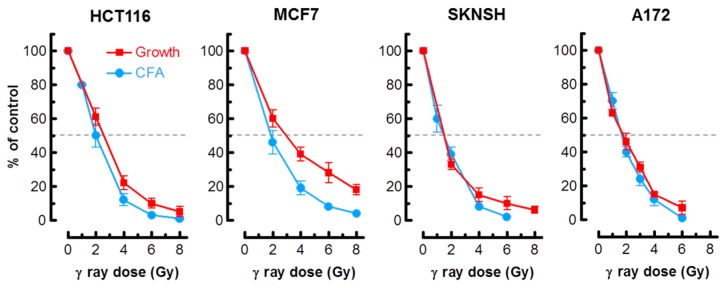
Radiosensitivity of the indicated cell lines evaluated by growth inhibition and colony formation assays. Growth inhibition and colony forming ability measurements were performed 3 days and 10 days post-irradiation, respectively. Bars, standard error (SE). CFA, colony forming ability.

As alluded to earlier, in most cell types, activation of the p53 pathway in response to moderate doses of ionizing radiation (e.g., 8 Gy) results in activation of early cell cycle checkpoints, suppression of apoptosis and sustained growth arrest through SIPS [2,3,4]. Consistent with these observations, radiosensitivity as measured by the colony formation assay in normal fibroblast strains and p53 wild-type solid tumor-derived cell lines has been shown to primarily reflect p21-dependent growth arrest through SIPS, and not cell death [2,3,6,7,20]. We predicted that the long-term clonogenic assay and the short-term (3 days post-irradiation) growth inhibition assay involving cell counting would indicate a comparable degree of radiosensitivity in p53 wild-type cells, given that the same p53 downstream effector (p21) is responsible for activating both early growth arrest (checkpoints) and sustained growth arrest (SIPS). The results presented in Figure 1 support this interpretation for the p53 wild-type lines MCF7, HCT116, SKNSH and A172. For each cell line, radiation doses resulting in 50% effect (Inhibiting Dose 50%; ID_50_) were comparable when measured by colony formation and growth inhibition assays.

Interestingly, we have recently reported that these two assays also indicate a comparable degree of radiosensitivity for solid tumor-derived cell lines that lack p21 or wild-type p53 activity [23]. Radiosensitivity in such cells was shown to reflect the creation of multinucleated giant cells, which remain viable and exhibit metabolic activity for extended periods (weeks) post-irradiation.

Although the colony formation assay is considered to be the “gold standard” for genotoxicity assessment, it has several drawbacks. Aside from being time consuming and labor intensive, preparation of good quality single-cell suspensions and evaluation of borderline macroscopic colonies are not trivial for some cell types, requiring considerable expertise. In addition, some solid tumor-derived cell lines (e.g., SUM52PE breast carcinoma) cannot be reliably evaluated by this assay because of their poor cloning efficiencies, and some cell lines (e.g., SKBR3 breast carcinoma) do not yield a good single-cell suspension by conventional approaches (e.g., after exposure to trypsin) [22].

**Figure 2:**
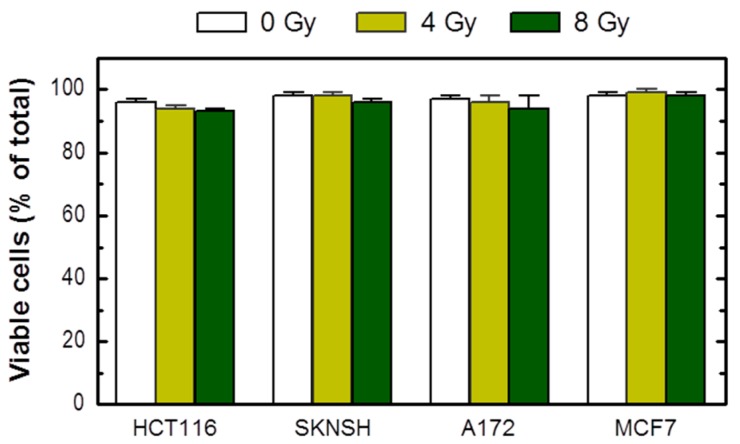
Viability of the indicated cell lines before (0 Gy) and 3 days after radiation exposure, evaluated by the trypan blue-exclusion assay. Bars, SE.

**Figure 3:**
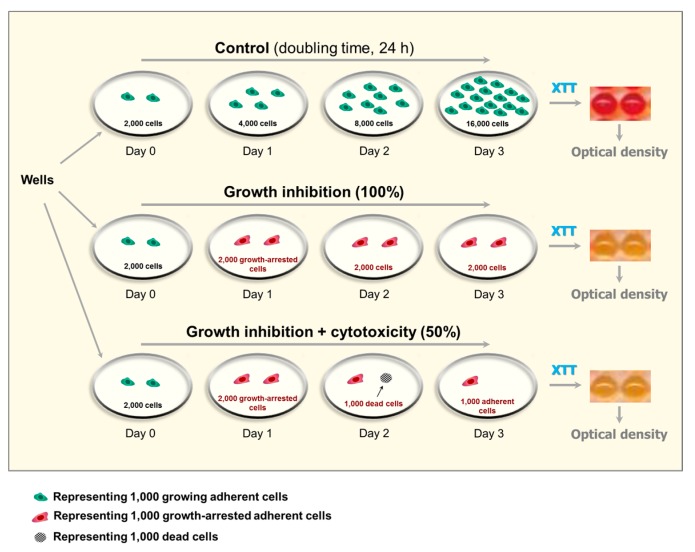
Depiction of anticipated outcome of multiwell plate colorimetric (XTT) assays after treatments that result in cytostatic (growth inhibition) effect only (e.g., 5 Gy of ionizing radiation), or cytostatic plus cytotoxic effects (e.g., >40 μM cisplatin). The example is given for a culture with a control population doubling time of 24 h. In this case, for “control” wells (upper left well), seeding 2000 cells per well (day 0) and incubating them with growth medium for 3 days (upper right well) will result in 16,000 cells/well at the time of optical density measurement. For “radiation” wells (middle left well) and “cisplatin” wells (lower left well), the number of cells per well is expected not to increase from day 0 to day 3, due to induction of growth arrest in virtually all cells (irradiation) or induction of growth arrest plus loss of viability (cisplatin treatment). It is important to note that most solid tumor-derived cell lines (e.g., HCT116) have shorter population doubling times than 24 h [22].

The growth inhibition assay circumvents these limitations. As pointed out previously [23], in addition to reproducibility and relative ease of performance, the growth inhibition assay, which is completed within 5 days of seeding the cells, enables one to utilize microscopic examination to track the long-term fate of adherent cells. To this end, cells in one set of dishes can be evaluated, either at the time of cell counting (i.e., 3 days after irradiation) or after further incubation, for different responses using a battery of single-cell methods. We routinely use one set of dishes for cell viability assessment by the trypan blue-exclusion assay under conditions that minimize the creation of false positives (e.g., cell detachment by exposure to trypsin) [23]. Based on this assay, we found that radiation exposure does not induce loss of viability (trypan blue staining) in cultures of these cell lines when determined at different times between 3 days (Figure 2) and 3 weeks (data not shown) post-irradiation.

### 2.2. Multiwell Plate Assays Markedly Underestimate Radiosensitivity

#### 2.2.1. Multiwell Plate Colorimetric/Fluorimetric Assays: Description

Short-term cell metabolic activity assays are widely used to assess radiosensitivity and chemosensitivity with solid tumor-derived cell lines [24,25,26,27]. Such assays are performed in a multiwell format and measure the ability of living cells to convert a detection reagent to its metabolite, often mediated by the mitochondrial oxidoreductases. The tetrazolium salts 3-(4,5-dimethylthiazol-2-yl)-2,5-diphenyl-tetrazolium bromide (MTT) and 2,3-bis-(2-methoxy-4-nitro-5-sulfophenyl)-2H-tetrazolium-5-carboxanilid (XTT) are the most commonly-used detection reagents in multiwell plate assays. Figure 3 summarizes the general steps for genotoxicity assessment by the XTT colorimetric assay.

In a typical assay, the cells are plated at “optimal” densities in the wells of a microtiter plate, exposed to a genotoxic agent, and incubated for 3 days. XTT (or MTT) is added to each well, and cultures are incubated for a few hours to allow the viable cells to covert the yellow tetrazolium salt (XTT or MTT) to its colored formazan derivative; XTT formazan is orange in color whereas MTT formazan is purple. The number of cells inoculated per well and the duration of cell incubation with XTT or MTT need to be optimized for each cell line.

MTT is readily taken up by the cells, but its formazan metabolite is water insoluble, requiring a solubilization step before data evaluation. XTT, on the other hand, requires an electron coupling step to facilitate its uptake by the cells, but the XTT metabolite is water soluble.

After these treatments, either with (MTT) or without (XTT) the metabolite solubilization step, the medium overlaying the control (sham-treated) cultures turns purple (MTT) or orange (XTT), as a result of conversion of the yellow tetrazolium salt to a colored metabolite by viable cells. Genotoxic treatment results in a decrease in color intensity of the medium, which is proportional to the number of viable cells/well at the time of incubation with XTT or MTT (Figure 3). The results are recorded in a plate reader, using absorbance measurements at 475 nm for XTT and 570 nm for MTT.

The CellTiter-Blue assay is also widely used for genotoxicity assessment. This assay is based on the ability of living cells to convert the redox dye resazurin (blue) into the resorufin product (pink). Resazurin has little intrinsic fluorescence until it is converted to its metabolite, which is highly fluorescent (579 nm excitation/584 nm emission). Thus, the CellTiter-Blue assay not only eliminates the need for electron coupling or metabolite solubilization steps required for tetrazolium-based assays, but it also enables fluorimetric evaluation of the data, which is considered to be more sensitive than absorbance measurements.

#### 2.2.2. Enlarged Cells Created Post-Irradiation Metabolize MTT

**Figure 4:**
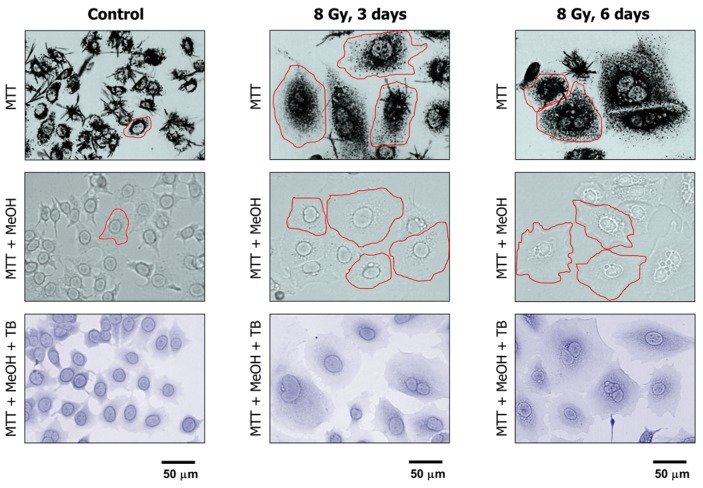
Representative bright-field microscopy images depicting the metabolic activity of MCF7 cultures before (control) and at indicated times after 8-Gy irradiation. Metabolic activity was measured by the ability of the cells to convert the yellow MTT to its purple formazan metabolite, appearing as dark granules and crystals. Upper row: images were acquired after incubation of cells with MTT for ~1 h. Middle row: following incubation with MTT, cells were fixed in methanol for 0.5 min to dissolve the MTT metabolite. The resulting purple medium was removed before acquiring images of cells. Lower row: MTT treated and methanol fixed cells were mildly stained with trypan blue (TB) to visualize their morphology. All images were acquired at the same magnification. The border of some cells is marked for clarity.

**Figure 5:**
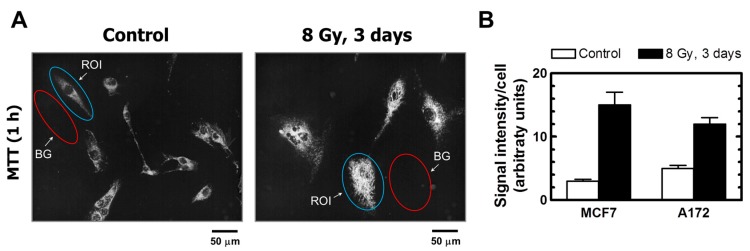
(**A**) Representative images of A172 cells used for image analysis. The images of MTT metabolites were acquired as described in Figure 4 legend (upper row). The images were then converted to grayscale and inverted. Blue and red ovals mark some regions of interest (reflecting MTT metabolites) and corresponding background regions used for image analysis, respectively. (**B**) Densitometric evaluation of MTT metabolic activity for the indicated cultures, expressed as signal intensity for regions of interest (cells) after corresponding background corrections. Mean values for at least 30 cells are presented for each sample. Bars, SE. ROI, region of interest; BG, background.

The properties of MTT make it particularly useful for microscopic assessment of individual cells in the absence of solvent treatment. To this end, cultures are incubated with medium containing MTT to allow viable cells to covert MTT to its purple, water-insoluble formazan. Under these conditions, MTT metabolites appear as intercellular formazan granules as well as needle-like formazan crystals [23]. We used this single-cell observation method to determine the metabolic activity of individual cells within cultures of the MCF7 and A172 cell lines before and at two time points (3 and 6 days) after exposure to ionizing radiation (8 Gy). Representative images are presented in Figure 4 and Figure 5A, and the results of image analysis are shown in Figure 5B. In these experiments, cells were incubated with medium containing MTT for ~1 h and their images were acquired (Figure 4, upper row; Figure 5A). Next, the medium was removed and the cells were treated with methanol to dissolve and remove intercellular tetrazolium granules and crystals; their images were acquired before (Figure 4, middle row) and after (Figure 4, lower row) mild staining with trypan blue. Two observations should be noted. First, enlarged cells that emerged in response to radiation exposure (i.e., cells undergoing or destined to undergo SIPS) showed a high degree of metabolic activity (Figure 4, upper row; Figure 5A). Second, the level of metabolic activity per cell (estimated from signal intensity in the “region of interest”) was markedly greater for irradiated cultures than for sham-irradiated controls (Figure 5B).

#### 2.2.3. Multiwell Plate Assays Lack Specificity

Several pitfalls of multiwell plate colorimetric assays have been extensively reviewed, including the impact of cell density and possible interference of test compounds with optical density measurements [28,29,30,31,32,33]. However, a fundamental caveat related to cell fate has been largely overlooked. In the MTT/XTT assays, for example, the change in optical density for control wells (purple or orange) versus treated wells is often assumed to reflect loss of viability consequent to genotoxic treatment. This interpretation would be tenable only if the number of cells at the time of assay evaluation (e.g., 3 days following genotoxic treatment) would be identical in control and treated wells; however, this is often not the case because genotoxic stress activates transient cell cycle checkpoints as well as a sustained proliferation block associated with, e.g., SIPS and/or creation of polyploid/multinucleated giant cells, which are characterized by maintenance of cell membrane integrity and ability to secrete growth-promoting factors (reviewed in [2,3,4]). Such growth-arrested cancer cells exhibit metabolic activity, including the ability to convert MTT to its purple formazan metabolite ([23]; Figure 4 and Figure 5A, and unpublished observations).

Thus, although many suppliers refer to these colorimetric assays in terms of “Cell Viability” or “Cytotoxicity,” all such assays that are performed in a multiwell plate format lack specificity and can lead to incorrect interpretations. As illustrated in Figure 3, multiwell plate colorimetric assays are expected to indicate comparable levels of genotoxicity for treatments that cause growth inhibition in the absence of cell killing (e.g., after exposure to moderate doses of ionizing radiation) and for treatments that induce loss of viability (e.g., after treatment with high concentrations of cisplatin).

#### 2.2.4. Radiosensitivity Measured by Multiwell Plate Assays in p53 Wild-Type Cell Lines

**Figure 6:**
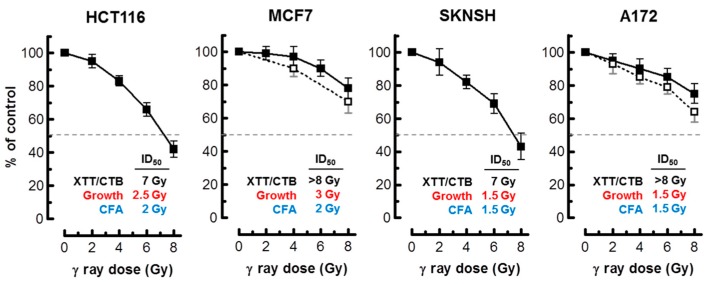
Radiosensitivity of the indicated cell lines evaluated by the 96-well plate XTT (solid squares) and CellTiter-Blue (open squares) assays. Bars, SE. The ID_50_ values for colorimetric (this Figure), growth inhibition and colony formation (Figure 1) assays are shown. CTB, CellTiter-Blue; CFA, colony forming ability; ID_50_, inhibiting dose 50%.

Assuming that radiation exposure would minimally alter the size of cancer cells, radiosensitivity as measured by multiwell plate assays would be expected to be comparable to that measured by growth inhibition and colony formation assays. This is not always the case, however; cancer cells undergoing SIPS following radiation exposure exhibit a markedly enlarged morphology. Sohn et al. [34], for example, reported that HCT116 cells exhibit nuclear localization of p21 and a dramatic increase in size at 48 h after irradiation. We made a similar observation with our panel of cell lines (data not shown). Accordingly, colorimetric assays are likely to underestimate radiosensitivity in p53 wild-type cell lines, given that highly enlarged cells created post-irradiation remain viable (e.g., Figure 2), and exhibit metabolic activity (e.g., Figure 4 and Figure 5). The results presented in Figure 6 are consistent with this notion. In MCF7 cultures, for example, the ID_50_ values measured by growth inhibition and multiwell plate assays (both measured 3 days post-irradiation) amounted to ~3 Gy and >8 Gy, respectively.

### 2.3. Cancer Cells Expressing Wild-Type p53 Do Not Readily Undergo Apoptosis within 3 Days Post-Irradiation

In most human cell types, activation of p53 signaling in response to genotoxic stress has been shown to supress (rather than promote) apoptosis [2,19]. Suppression of apoptosis by p53 signaling has been attributed not only to p21, which acts at different levels of the death cascade, but also to p53-dependent expression of numerous anti-apoptotic proteins including 14-3-3δ, WIP1 (wild-type p53-induced phosphatase 1), and DNAJB9 (DNAJ homolog subfamily B member 9) [19]. Consistent with these observations, p53 wild-type cancer cell lines such as HCT116 [34,35], MCF7 [6,35] and A172 [36] are markedly refractory to undergoing apoptosis in response to ionizing radiation.

**Figure 7:**
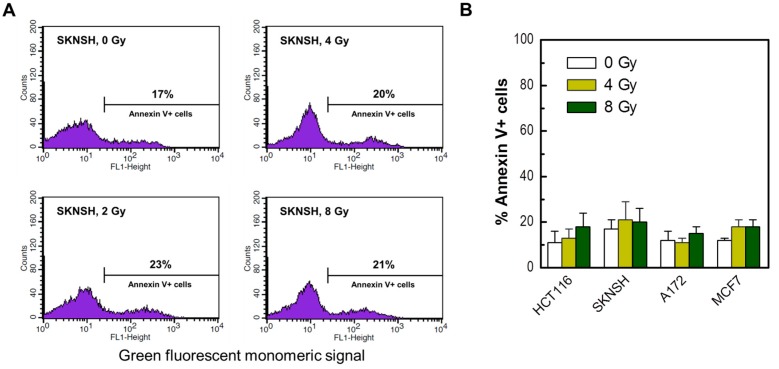
(**A**) Representative flow cytometry profiles of SKNSH cells stained with Annexin V (FITC) before and 3 days after irradiation. (**B**) The percentages of Annexin V-positive cells in the indicated cell lines before and 3 days after irradiation. Bars, SE.

Apoptosis in our previous work was evaluated by microscopic examination of Hoechst 33342-stained cells for morphological criteria (nuclear condensation and fragmentation). We sought to verify these observations by flow cytometric determination of Annexin V-positive (phosphatidylserine-externalized) cells. The results are presented in Figure 7. In these experiments, cultures of the four cancer cell lines used in the radiosensitivity studies were exposed to radiation and incubated for 3 days. The corresponding floating and adherent cells were combined and evaluated by flow cytometry. As expected, radiation exposure resulted in apoptosis (Annexin V staining) in only a small fraction of these p53 wild-type cells.

**Figure 8:**
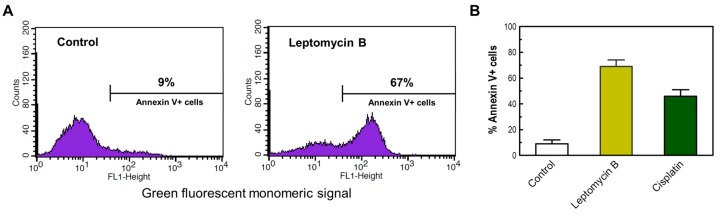
(**A**) Representative flow cytometry profiles of HCT116 cells assessed for Annexin V positivity after incubation in the absence (control) or presence of leptomycin B (2 μM) for 3 days. (**B**) The percentages of Annexin V-positive cells in HCT116 cultures incubated in the absence or presence of leptomycin B (2 μM) or cisplatin (60 μM) for 3 days. Bars, SE.

As a positive control, we also determined the apoptotic response of selected cancer cell lines (e.g., HCT116) following incubation with high concentrations of the chemotherapeutic drug cisplatin or the nuclear export inhibitor leptomcyin B. Treatment with cisplatin (60 μM) or leptomycin B (2 μM) for 3 days resulted in apoptosis in a high proportion of HCT116 cells when evaluated by Annexin V/flow cytometry (Figure 8) and morphological criteria (data not shown).

## 3. Materials and Methods

### 3.1. Cells and Culture Conditions

The four cancer cell lines used in the present study were purchased from the American Type Culture Collection (Rockville, MD, USA). Cells were cultured as monolayers in DMEM/F12 nutrient medium supplemented with 5% (*v*/*v*) fetal bovine serum as described in [7]. All cultures were free of *Mycoplasma* contamination.

### 3.2. Reagents

The vital dye trypan blue (Sigma, St. Louis, MO, USA), the CellTiter-Blue reagent (Promega, Madison, WI, USA) and the tetrazolium dyes MTT and XTT (Roche Diagnostics, Penzberg, Germany) were used as recommended by the manufacturers.

### 3.3. Radiation Exposure

Exposure to ^60^Co γ-rays was performed in a Gammacell 220 unit as described [37].

### 3.4. Radiosensitivity Assays

Radiosensitivity evaluation by growth inhibition, colony formation and 96-well plate assays was performed as described [23]. Flow cytometric assessment of Annexin V-positive cells was performed as described [38].

## 4. Conclusions

In the current study, we have demonstrated that the conventional growth inhibition assay generates radiosensitivity data comparable to that obtained by the slower and more technically challenging colony formation assay for p53 wild-type cancer cell lines. On the other hand, the response measured by multiwell plate colorimetric/fluorimetric assays is markedly skewed towards radioresistance, which we assume to reflect the emergence of highly enlarged, growth-arrested and viable cells post-irradiation (i.e., cells undergoing SIPS). In addition, we have confirmed that exposure to moderate (“clonogenic survival-curve-range”) doses of ionizing radiation does not induce apoptosis (as judged by the Annexin V/flow cytometry approach) or loss of viability in the p53 wild-type cancer cell lines that we examined. These observations, in concert with those recently reported by us for p53 null or mutant p53-expressing cancer cell lines [23], give credence to the caution advised by the Nomenclature Committee on Cell Death [39] and others [40] with regard to the potential for misinterpreting the outcome of cell-based genotoxicity data in terms of loss of viability and hence cell death.

Short-term multiwell plate assays are indispensable for high throughput studies, e.g., screening compound libraries for genotoxicity (proliferation block and/or cell death) towards the US NCI panel of cancer cell lines. However, it is becoming increasingly evident that the effect measured by such assays primarily reflects growth inhibition and not loss of viability [3,19,23]. Our current studies with p53 wild-type cancer cell lines exposed to moderate doses of ionizing radiation provide further support for this conclusion.

## Abbreviations

SIPS	Stress-induced premature senescence
CFA	Colony forming ability
DNAJB9	DNAJ homolog subfamily B member 9
WIP1	Wild-type p53-induced phosphatase 1
CDK	Cyclin dependent kinase
MTT	3-(4,5-dimethylthiazol-2-yl)-2,5-diphenyl-tetrazolium bromide
XTT	2,3-bis-(2-methoxy-4-nitro-5-sulfophenyl)-2H-tetrazolium-5-carboxanilid
CTB	CellTiter-Blue
ID_50_	Inhibiting dose, 50%
SE	Standard error
TB	Trypan blue
ROI	Region of interest
BG	Background
